# Vitamin D Deficiency Impacts Exposure and Response of Pravastatin in Male Rats by Altering Hepatic OATPs

**DOI:** 10.3389/fphar.2022.841954

**Published:** 2022-02-17

**Authors:** Jinfu Peng, Guoping Yang, Zhijun Huang

**Affiliations:** ^1^ Department of Pharmacy, The Third Xiangya Hospital, Central South University, Changsha, China; ^2^ Center for Clinical Pharmacology, The Third Xiangya Hospital, Central South University, Changsha, China; ^3^ Department of Nephrology, The Third Xiangya Hospital, Central South University, Changsha, China

**Keywords:** VD deficiency, OATPs, pravastatin, pharmacokinetics, pharmacodynamic

## Abstract

This study aimed to determine the effect of vitamin D (VD) deficiency on the efficacy and pharmacokinetics of pravastatin and clarify whether the effects are mediated by Organic anion-transporting polypeptides (OATPs). Experiments were conducted in rats to explore the effect of VD deficiency on the pharmacodynamics and pharmacokinetics of pravastatin. In the pharmacodynamic study, rats were fed a VD-free or VD-supplement high-fat diet for 25–30 days, and plasma 25(OH)VD was dynamically monitored. The response of pravastatin (changes in blood lipids) on rats were then examined after 15 days of pravastatin treatment. In the pharmacokinetic study, rats were fed a VD-free or VD-supplement diet for 25–30 days. The pharmacokinetics of single oral dose pravastatin was then studied, and intestinal and hepatic Oatp1a1 and Oatp2b1 expression was determined using quantitative polymerase chain reaction (qPCR) and western blot. Furthermore, OATP1B1 and OATP2B1 expression in Huh7 cells with or without 1.25(OH)_2_D were assessed via qPCR and western blot. For the pharmacodynamic study, the decrease of total cholesterol and increase of high-density lipoprotein cholesterol in VD-deficient rats were smaller than in VD-sufficient rats, indicating that VD deficiency reduced the response of pravastatin in rats. For the pharmacokinetic study, the plasma exposure slightly increased, and liver exposure decreased in VD-deficient rats, but not significantly. VD deficiency decreased the Oatp1a1 and Oatp2b1 expression in the liver, but not in the small intestine. Similarly, OATP1B1 and OATP2B1 protein levels in Huh7 cells were reduced when 1.25(OH)_2_D was absent. In conclusion, VD deficiency can decrease the response of pravastatin in rats by reducing the liver pravastatin exposure and expression of hepatic OATPs, consistent with the extended hepatic clearance model theory.

## Introduction

Vitamin D (VD) is a steroid derivative, which exists in different forms, including vitamin D_2_ (ergocalciferol) and vitamin D_3_ (cholecalciferol). It is converted to 25-hydroxyvitamin D (25(OH)D) in the liver and transformed into the activated form (1,25-dihydroxyvitamin D, 1.25(OH)_2_D) in the kidney (Dusso et al., 2005). VD deficiency [25(OH)D < 20 ng/ml (50 nmol/L)] (Naeem, 2010) is common in the general population, especially among the elderly, pregnant women, children, and long-term bedridden patients (Mehboobali et al., 2015; Pagliardini et al., 2015; Riaz et al., 2016). VD deficiency can cause muscular and skeletal diseases (6) and affect the immune, nervous, and cardiovascular systems (Looker et al., 2002; Lee et al., 2008; Christakos et al., 2013). VD can also influence the efficacy of drugs (Peng et al., 2020) and induce expression of metabolic enzymes (Schmiedlin-Ren et al., 1997; Drocourt et al., 2002; Echchgadda et al., 2004) and drug transporters (Fan et al., 2009; Kim et al., 2014; Claro da Silva et al., 2016; Quach et al., 2018). It was reported that VD deficiency affected the efficacy and adverse effects of Cytochrome P450 (CYP) enzyme substrate drugs, e.g., atorvastatin, simvastatin, lovastatin, fluvastatin, pitavastatin (Schwartz, 2009; Pérez-Castrillón et al., 2010; Michalska-Kasiczak et al., 2015; Qin et al., 2015; Geng-ke et al., 2017), and rosuvastatin (Qin et al., 2015; Hileman et al., 2017; Chogtu et al., 2020). However, the mechanisms involved are not clear.

Pravastatin is widely used to treat hypercholesterolemia and prevent cardiovascular disease. It is not metabolized by enzymes, is mainly excreted through bile, and is commonly used as a probe drug of organic anion-transporting polypeptide (human OATP/rat Oatp) (Komai et al., 1992; Sirtori, 2014). Oatp2b1 (Slco2b1) and Oatp1a1 mediates the intestinal and hepatic uptake of pravastatin in rats (Hsiang et al., 1999; Tokui et al., 1999; Kobayashi et al., 2003; Sasaki et al., 2004; Sakamoto et al., 2008; Shirasaka et al., 2011; Keiser et al., 2017). In human, OATP2B1 contributes to the intestinal absorption of pravastatin into the blood circulation, while OATP1B1 is localized in the liver and pravastatin is taken up there (Keiser et al., 2017; Izumi et al., 2018). Thereafter, biliary excretion and renal secretion of pravastatin occur via multidrug resistance-associated proteins (MRP2/Mrp2) (Itagaki et al., 2008; Watanabe M. et al., 2011; Ellis et al., 2013) and organic anion transporter 3 (OAT3/Oat3) (Maki et al., 2002; Khamdang et al., 2004; Watanabe T. et al., 2011; Mathialagan et al., 2017), respectively. By impairing the function of the intestinal OATP2B1/Oatp2b1, both the plasma and hepatic drug exposure are reduced; if only the function of hepatic OATPs/Oatps are reduced, the plasma exposure will increase, while the hepatic exposure or drug concentration-time profile will change (Ieiri et al., 2009; Patilea-Vrana and Unadkat, 2016). In this study, we will investigate the effect of VD deficiency on the pharmacokinetics and pharmacodynamics of the OATPs probe drug pravastatin and the expression of OATPs.

Clarifying the effect of VD deficiency on pravastatin and OATPs is important for several reasons. First, it can uncover the mechanism of VD deficiency induced changes in the efficacy and adverse reactions of statins. Second, it can provide a theoretical basis for the rational use of pravastatin and other OATP substrate drugs (e.g., other statins, digoxin) in VD-deficient patients, which helps patients avoid medication risks. Third, it can suggest whether VD deficiency needs to be considered in clinical studies of drugs, especially OATP substrate drugs. Last, it can indicate new ways of using VD, for example, as an adjunct to disease treatment. In this study, a rat model of VD deficiency was established. Animal and cell experiments were consequently conducted to investigate the efficacy and exposure of pravastatin, and expression of intestinal and hepatic OATPs/Oatps under VD deficiency.

## Materials and Methods

### Animal and Study Diet

Sprague Dawley rats (SD rats, male, 150–160 g, 5–7 weeks) were sourced from Hunan STA laboratory animal Co., Ltd. Study diets included VD_3_-supplement (4.4 UI/g, 0.11 ug/g) and VD-free feeds (Co60 sterilized feed) from Beijing KEAO XIELI Feed Co., Ltd., stored at –80°C. The ingredients of the VD-supplement and VD-free feeds are shown in the Supplementary Table S1.

### Pharmacodynamic and Pharmacokinetic Experiments in Rats

**FIGURE 1:**
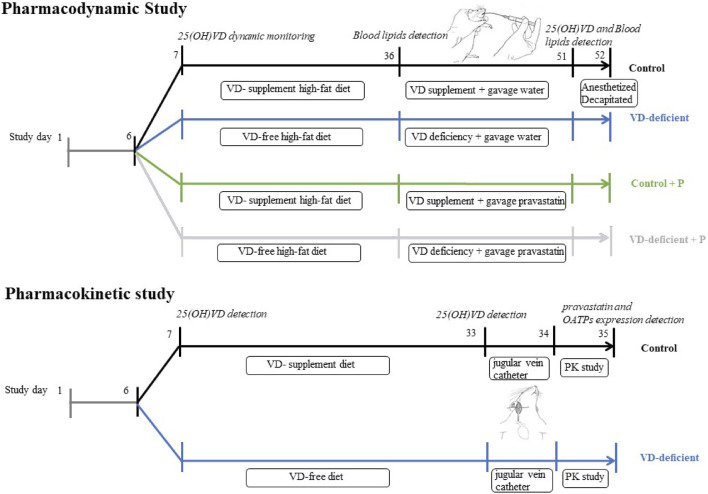
Study design of pharmacodynamic and pharmacokinetic experiments in rats. Pharmacodynamic study: pharmacodynamic study of pravastatin in VD-sufficient (Control, Control + P) or VD-deficient (VD-deficient, VD-deficient + P) rats. Pharmacokinetic study: pharmacokinetic study of pravastatin in VD-sufficient (Control) or VD-deficient rats. Control: rats fed on a VD-supplement diet with or without gavage of water; VD-deficient: rats fed on a VD-free diet with or without gavage of water; Control + P: rats fed on a VD-supplement diet and administered pravastatin via gavage for 15 days; VD-deficient + P: rats fed on a VD-free diet and administered pravastatin *via* gavage for 15 days.

Approval for this study was issued by the Medical Ethics Committee of the Third Xiangya Hospital of Central South University (No: 2015-S126). Experiments performed on rats followed the National Institutes of Health guide for the care and use of laboratory animals (eighth edition). The experiments in rats were implemented as summarized in Figure 1. Briefly (but detailed below), rats for pharmacodynamic study were fed on a VD-free/supplement high-fat diet for 25–30 days, followed by gavage of water/pravastatin for 15 days; plasma 25(OH)VD levels were dynamically monitored, and blood lipid was measured before and after gavage of water/pravastatin (A08D8L50107, 98%, Shanghai Yuanye Bio-technology Co., Ltd.). On the other hand, rats for pharmacokinetic study were fed on a VD-free/supplement diet for 25–30 days, plasma 25(OH)VD were measured, a single dose of pravastatin was then given by gavage, and plasma pravastatin and OATPs expression were determined.

### Effect of VD Deficiency on the Pravastatin Efficacy in Rats (Pharmacodynamic Study)

The study design is shown in Figure 1 (Pharmacodynamic study). SD rats (150–160 g, 5–7 weeks) were fed with normal feed for 1 week, to adapt to the environment, and plasma 25(OH)VD levels were measured using LC-MS/MS (Supplementary File S1). Thereafter, they were randomly divided into four groups: Control (VD-supplement high-fat diet followed by gavage of water), VD-deficient (VD-free high-fat diet followed by gavage of water), Control + P (VD-supplement high-fat diet followed by gavage of pravastatin), and VD-deficient + P (VD-free high-fat diet followed by gavage of pravastatin). Each group had nine rats. A 2 ml sample of blood was drawn from the tail vein before feeding and at 5, 15, and 25 days to assess the plasma 25(OH)VD_2_ and 25(OH)VD_3_. Rats in the VD-deficient groups (VD-deficient, VD-deficient + P) were fed on a VD-free high-fat diet, and those in the control groups (Control, Control + P) were fed on a VD-supplement high-fat diet (4.4 UI/g feed, 0.11 ug/g), then kept in a dark environment for 25–30 days and plasma 25(OH)VD levels were dynamically monitored. Thereafter, considering the 25(OH)VD concentration in healthy humans (20–100 ng/ml) and normal SD rats (32.6 ± 6.78 ng/ml), rats in the control groups (Control, Control + P) with 25(OH)VD > 100 ng/ml were excluded, and rats in the VD-deficient groups (VD-deficient, VD-deficient + P) with 25(OH)VD > 20 ng/ml were excluded.

After 25–30 days, blood was drawn from the tail vein, the lipid (TG, TC, HDL-C) in the blood was measured using the relevant kits (Beijing Leadman Biochemical Co., Ltd., 711032K). Then, pravastatin (10 mg/kg/day, equivalent to 80 mg/d for human, Control + P and VD-deficient + P) or an equal volume of water (Control and VD-deficient) was then administered to the rats using gavage for 15 days, based on body weight. On the morning of the last day, rats were lightly anesthetized with carbon dioxide and decapitated, after starving for 12 h, and blood was collected to examine for lipid content and 25(OH)VD after treatment. The rate of change in blood lipids (percentage of blood lipid change) was calculated using Eq. 1, to compare the lipid-lowering effect of pravastatin between the Control groups and the VD-deficient groups.

$$percentage\;of\;blood\;lipid\;change\left(\%\right)=\;\frac{blood\;lipid\;after\;treatment\;-\;blood\;lipid\;before\;treatment}{blood\;lipid\;before\;treatment}$$ (1)

### Effect of VD Deficiency on the Pravastatin Pharmacokinetics in Rats (Pharmacokinetic Study)

The study design is shown in Figure 1 (Pharmacokinetic study). SD rats were randomly divided into a Control group (VD-supplement diet followed by gavage of a single dose of pravastatin) and a VD-deficient group (VD-free diet followed by gavage of a single dose of pravastatin). A jugular vein catheter was inserted into the rat on the 26th day and plasma 25(OH)VD levels were measured. A single dose (pravastatin dose of 20 mg/kg) was administered using gavage after 12 h of fasting. Blood samples were collected at 0, 10, 30, 45, 60 min, 2 h, and 4 h after administration, and the rats were then euthanized. Liver and small intestine tissues were collected, and mRNA and Oatp2b1 and Oatp1a1 protein levels were determined using quantitative polymerase chain reaction (qPCR) and western blot (WB), respectively. The blood samples were centrifuged to obtain plasma samples, which were stored at −20°C. The concentrations of pravastatin in plasma and liver tissue were determined using LC-MS/MS (Supplementary File S1). The noncompartmental PK Analysis (NCA) module in WinNonlin 8.2.0 software was used to assess the pharmacokinetic parameters [area under the curve from the time of dosing to the last measurable concentration and extrapolated to infinity (AUC_inf_) and maximum plasma concentration (C_max_)] of pravastatin in the plasma of each rat.

### Cell Experiments

Huh7 cells (Shanghai GeneChem Technology Co., Ltd.) were cultured in 10% MEM medium, and the medium was changed every 2–3 days. The logarithmic growth cells were plated into groups (6-well plate). Blank control (ethanol), 50 nM 1.25(OH)_2_D_3_ (Aladdin, K2005014) or 500 nM 1.25(OH)_2_D_3_ were added when the plated confluence rate was about 60–70%. After incubating for 72 h, the cells were collected, and mRNA and OATP2B1 and OATP1B1 protein levels were then assessed using qPCR and WB, respectively.

### qPCR for Rat Slco2b1 and Slc1a1, and Human SLCO2B1 mRNA and SLCO1B1

In the pharmacokinetic study, about 0.02 g of the tissue was taken, 1 ml of Trizol (Thermo, 15596026) was added, and then the homogenate was thoroughly ground. For cells, the procedure was similar to our previous report (Peng et al., 2015): 1 ml/well of Trizol was added and the cells were fully pipetted and mixed, before the mRNA was then extracted. After that, concentration and purity were determined using an ultraviolet spectrophotometer. With total mRNA as the template, complementary DNA (cDNA) reverse transcription and real-time qPCR were performed using a kit (Beijing CWbio Company, CW2569). Primers were listed as shown below. In this study, β-actin was the internal control used to assess the rat tissue samples, and GAPDH (Glyceraldehyde-3-Phosphate Dehydrogenase) was used for cell samples. The relative expression differences between the target gene and the internal control were compared using the 2^−△△Ct^ method. Primers:

**Table:** 

Rat-β-actin	Human-GAPDH
β-actin-F: ACA​TCC​GTA​AAG​ACC​TCT​ATG​CC	GAPDH-F ACA​GCC​TCA​AGA​TCA​TCA​GC
β-actin-R: TAC​TCC​TGC​TTG​CTG​ATC​CAC	GAPDH-R GGT​CAT​GAG​TCC​TTC​CAC​GAT
Rat-Slco1a1	Human-SLCO1B1
Slco1a1-F: GTG​ACC​CCC​ACA​CTA​CAC​TT	SLCO1B1-F: ATT​CTC​GAT​GGG​TTG​GAG​CT
Slco1a1R: TCA​GCT​CTA​AAT​ACT​TCC​AAC​TGT​G	SLCO1B1-R: TTT​GGA​GTT​TGG​GGC​AAG​AA
Rat-Slco2b1	Human-SLCO2B1
Slco2b1-F: GCC​ACC​TTC​CTG​CCT​AAG​TTC​C	SLCO2B1-F: GCC​TGC​CGC​TCT​TCT​TAT​C
Slco2b1-R: TAG​CAG​ACA​CAA​CAG​CGA​CCC	SLCO2B1-R: GGT​TAA​AGC​CGT​CCA​ATG​GG

### Western Blot for Rat Oat2b1 and Oatp1a1, and Human OATP2B1 and OATP1B1

Proteins were measured as previously reported (Peng et al., 2015). Briefly, tissue in the pharmacokinetic study weighing 0.016 g was cut, washed with ice-cold PBS, and repeatedly ground in the homogenizer with 170 µL RIPA lysis solution (Shanghai Beyotime Biotechnology Company, P0013B, with added protease inhibitor) until no tissue block was visible. For cells, 250 µL lysis buffer was added to each well, the plates were put on ice for 10 min and then centrifuged at 12,000 rpm for 15 min (4°C). Centrifuged supernatant was transferred into a 0.5 ml centrifuge tube. Proteins (40 µg) were separated by electrophoresis and transferred to a PVC membrane. A certain proportion of primary antibody was diluted with 1*TBST [Oatp1a1 (1:2000, bs-3913R, Bioss Co., Ltd.), OATP2B1/Oatp2b1 (1:2000, bs-3913R, Bioss Co., Ltd.), OATP1B1 (1:750, abs134836, Abison Biotechnology Co., Ltd.), β-actin or GAPDH (1:5,000, Proteintech)], HRP-labelled secondary antibody (Proteintech), and membrane were incubated. Then the membranes were washed and incubated with chemiluminescence solution (US Advansta, K-12045-D50) for 3 min, exposed in the dark box for 5 min, and developed. ImageJ software was used to determine the gray value. The gray value ratio of the target gene and the internal reference gene was used for statistical analysis.

### Statistical Analysis

All data were expressed as mean ± SD. GraphPad Prism 8.3 software was used for graphing and statistical analysis. An unpaired t-test was used to assess statistical significance between the two groups. One-way analysis of variance (ANOVA) and Tukey’s multiple comparison tests were conducted to evaluate multiple comparisons. *p* < 0.05 was considered as statistically significant.

## Results

### Dynamic Monitoring of Growth and Plasma 25(OH)VD of Rats

The body weight of rats fed on a VD-free or VD-supplement diet (not high-fat) (pharmacokinetic study, Supplementary Figure S1A) was lower than that of rats fed on a VD-free or VD-supplement high-fat diet (pharmacodynamic study, Supplementary Figure S1D). There was no difference of water and food consuming between Control group and VD-deficient group (Supplementary Figures S1B,C,E,F). Long-term intragastric administration of pravastatin alleviated the weight gain, while VD deficiency had no significant effect on body weight (Supplementary Figures S1A,D).

**FIGURE 2:**
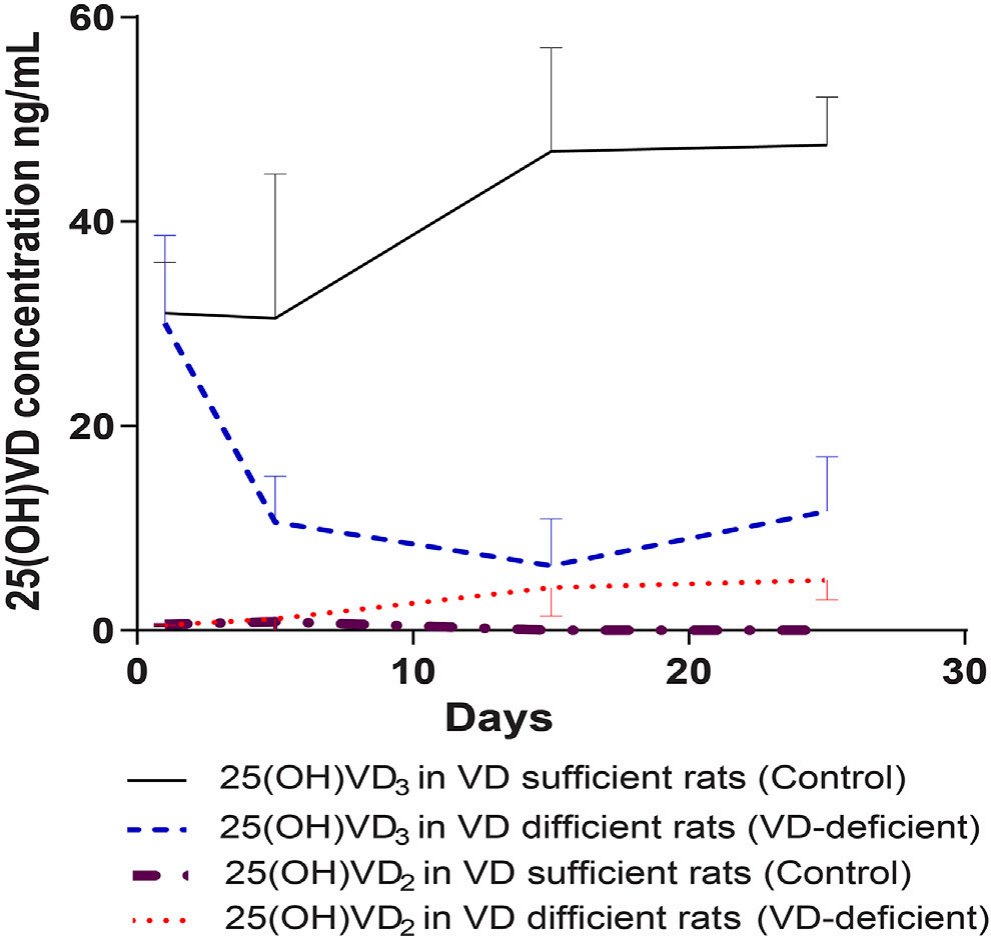
Dynamic monitoring of plasma 25(OH)VD3 and 25(OH)VD2 in rats in pharmacodynamic study. Control: rats fed on a VD-supplement high-fat diet for 25–30 days (*n* = 14), plasma 25(OH)VD concentration is 20–100 ng/ml; VD-deficient: rats fed on a VD-free high-fat diet for 25–30 days (*n* = 16), plasma 25(OH)VD concentration is <20 ng/ml; Total 25(OH)VD: the total 25(OH)VD_2_ and 25(OH)VD_3_ concentrations in plasma.

The normal 25(OH)VD_3_ concentration of the rats in the pharmacodynamic study, before the experiments, was 30.6 ± 6.78 ng/ml, and 25(OH)VD_2_ was less than 2 ng/ml. At the same initial concentration (*p* = 0.642), 25(OH)VD_3_ and 25(OH)VD_2_ reached steady state after 2 weeks of feeding: 25(OH)VD_2_ was almost reduced to 0 in the control group (*n* = 14) and increased to 4.94 ± 1.92 ng/ml in the VD-deficient rats (*n* = 16), while 25(OH) VD_3_ was 47.48 ± 4.73 ng/ml in the control and 11.65 ± 5.36 ng/ml in VD-deficient rats (Figure 2). Overall, two rats in the control groups had 25(OH)VD concentrations greater than 100 ng/ml, and four rats in the VD-deficient groups had concentrations greater than 20 ng/ml. These rats were excluded from the analysis and from the following study.

### Vitamin D Deficiency Decreases the Pravastatin Response in Rats (Pharmacodynamic Study)

**FIGURE 3:**
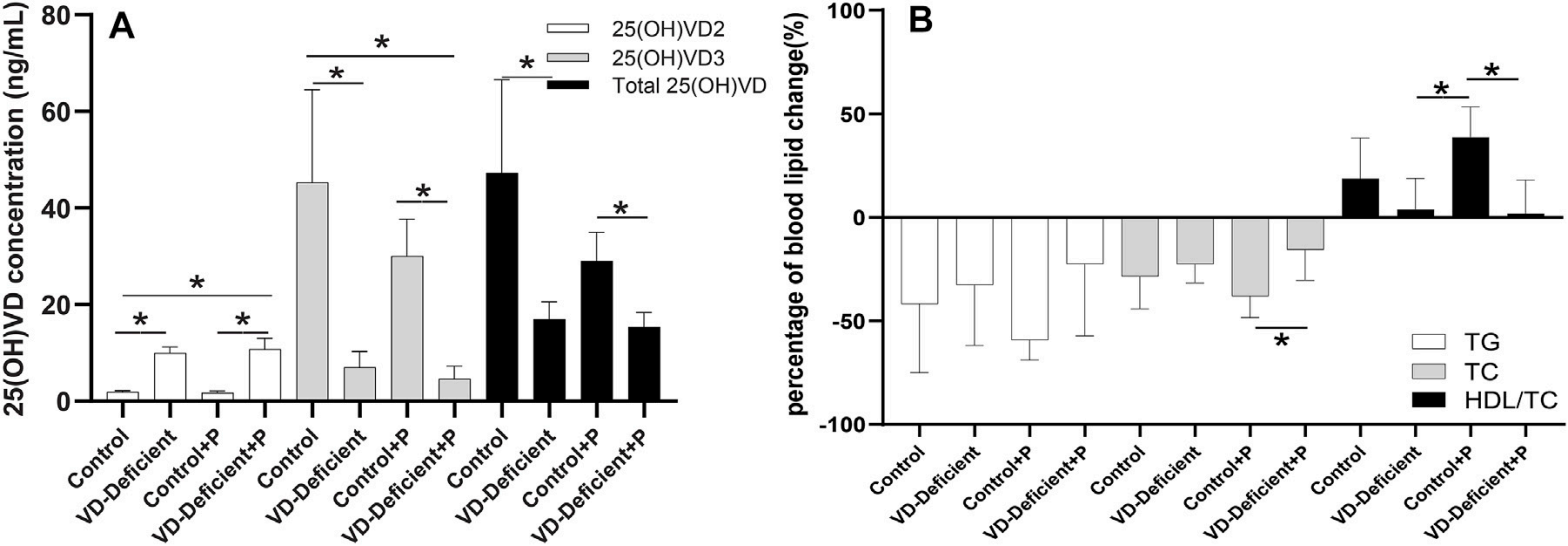
Effect of vitamin D deficiency on the pravastatin efficacy in rats. **(A)** Final 25(OH)VD_2_, 25(OH)VD_3_ and total 25(OH)VD concentration in rats fed on a VD-supplement/free high-fat diet with/without pravastatin. 25(OH)VD_3_ and total 25(OH)VD in VD-sufficient rats were higher than VD-deficient rats, and 25(OH)VD_2_ was lower (*p* < 0.05). **(B)** Percentage of blood lipid change (TG, TC, and HDL) in rats fed on a VD-supplement/free high-fat diet with/without pravastatin. Among the four groups, TG and TC levels in rats fed on a VD-supplementation diet and treated with pravastatin (Control + P) decreased the most, whereas HDL-C/TC increased the most. TG and TC levels in VD-sufficient rats were lower than in VD-deficient rats, while HDL-C/TC was higher. Control: rats fed on a VD-supplement high-fat diet (*n* = 7); VD-deficient: rats fed on a VD-free high-fat diet (*n* = 7); Control + P: rats fed on a VD-supplement high-fat diet and administered pravastatin via gavage for 15 days (*n* = 7); VD-deficient + P: rats fed on a VD-free high-fat diet and administered pravastatin via gavage for 15 days (*n* = 9). TG: triglycerides, TC: total cholesterol, HDL-C: high-density lipoprotein cholesterol. Y-axis: Percentage of blood lipid change = (blood lipid 15 days after administration—blood lipid before administration)/blood lipid before administration, *p < 0.05: statistically significant difference, using ANOVA followed by Tukey’s post-hoc test.

After the pharmacodynamic study, rats in the control groups had 25(OH)VD concentrations of 20–100 ng/ml (Control: 47.21 ± 19.33 ng/ml, *n* = 7; Control + P: 29.04 ± 5.90 ng/ml, *n* = 9), which was close to the VD level of normal rats, while rats in the VD-deficient groups had less than 20 ng/ml (VD-deficient: 16.99 ± 3.60 ng/ml, *n* = 7; VD-deficient + P: 15.36 ± 2.98 ng/ml, *n* = 7) (Figure 3A).

There was no difference in blood lipid level (TG: *p* = 0.591; TC: *p* = 0.403; HDL-C/TC: *p* = 0.137) between Control and VD-deficient groups without pravastatin, although the TG and TC decreased less, and HDL increased less in the VD-deficient rats. When administering pravastatin, the decrease of TC in the VD-deficient rats was lower than that of the VD-sufficient rats (VD-deficient + P vs Control + P: TC: −15.52% vs. −38.16%, *p* = 0.032), and HDL-C/TC increased less (1.86 vs. 38.82%, *p* = 0.005) (Table 1; Figure 3B). Among the four groups, the TG and TC of rats in the Control + P group decreased the most, and HDL-C/TC increased the most (Table 1; Figure 3B). Furthermore, the pravastatin-induced change of TG, TC and HDL/TC in the VD-sufficient rats (Control + P subtracting Control) were -17.40%, -9.62%, and 20.09%, respectively, while values in the VD-deficient groups (VD-deficient + P subtracting VD-deficient) were 10.10%, 7.06%, and −1.90%, respectively, which indicated that the effects of pravastatin were greater in the Control groups (VD-sufficient rats).

**TABLE 1:** The effect of vitamin D deficiency on the lipid-lowering efficacy of pravastatin (percentage of blood lipid change) in rats.

		TG				TC				HDL-C/TC			
Group		Control	VD-deficient	Control + P	VD-deficient + P	Control	VD-deficient	Control + P	VD-deficient + P	Control	VD-deficient	Control + P	VD-deficient + P
*N*		7	7	7	9	7	7	7	9	7	7	7	9
percentage of blood lipid change (%)	Mean	−41.84	−32.60	−59.24	−22.51	−28.54	−22.58	−38.16	−15.52	18.73	3.76	38.82	1.86
	SD	33.18	29.32	9.70	34.75	15.74	9.06	10.27	14.83	19.70	15.13	14.53	16.23
	P	0.93		0.18		0.82		0.03		0.36		0.005	

TG: triglycerides, TC: total cholesterol, HDL-C: high-density lipoprotein cholesterol. N: number of rats. Control: rats fed on VD-supplement high-fat diet (*n* = 7), plasma 25(OH)VD, concentration is 20–100 ng/ml; VD, deficient: rats fed on VD-free high-fat diet (*n* = 7), plasma 25(OH)VD, concentration is <20 ng/ml; Control + P: rats fed on VD-supplement high-fat diet and administrated pravastatin via gavage for 15 days (*n* = 7); VD -deficient + P: rats fed on VD-free high-fat diet and administrated pravastatin *via* gavage for 15 days (*n* = 9). Total 25(OH)VD: the total 25(OH)VD_2_ and 25(OH)VD_3_ concentrations in plasma. Percentage of blood lipid change = (blood lipid 15 days after administration-blood lipid before administration)/blood lipid before administration). *p* < 0.05: statistically significant difference, ANOVA, followed by Tukey’s post-hoc test.

### Effect of Vitamin D Deficiency on the Pravastatin Pharmacokinetics (Pharmacokinetic Study)

**FIGURE 4:**
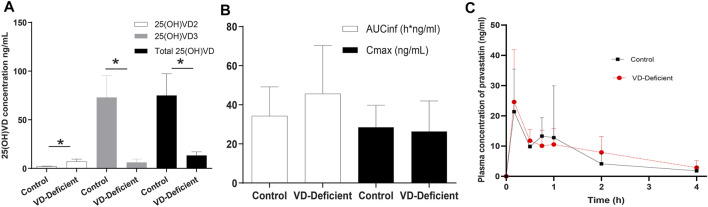
Pravastatin pharmacokinetic study results in rats. **(A)** Plasma 25(OH)VD concentration after feeding for 25 days with a VD-supplement diet (Control group, *n* = 6) or a VD-free diet (VD-deficient, *n* = 9). Plasma 25(OH)VD concentration in the deficient group fed on a VD_3_-free diet was lower than that of the control group (13.2 ± 6.26 ng/ml vs. 74.98 ± 22.30 ng/ml). **(B)** Pravastatin pharmacokinetics parameters. AUC_inf_ in the VD-deficient group is slightly higher, but both AUC_inf_ and C_max_ are not significantly different in the control and VD-deficient group. **(C)** Pravastatin time profile concentration of control and deficient groups. AUC_inf_: area under the curve from the time of dosing extrapolated to infinity, C_max_: maximum plasma concentration. *p* < 0.05: statistically significant difference, using two-tailed unpaired t-test.

There were six and nine rats with sufficient and deficient VD, respectively (three rats with excess VD concentration were not included in the statistical analysis). The total 25(OH)VD concentrations (mean ± SD) in the Control and VD-deficient groups were 74.98 ± 22.30 ng/ml and 13.2 ± 6.26 ng/ml, respectively (Figure 4A). These rats were intubated in the jugular vein and were used for pharmacokinetic studies.

The results showed that the C_max_ of the Control (*n* = 6) and VD-deficient rats (*n* = 9) were 28.53 ± 11.33 ng/ml and 26.40 ± 15.59 ng/ml, respectively, and the AUC_inf_ were 34.28 ± 14.86 h*ng/mL and 45.63 ± 24.68 h*ng/mL, respectively. Although the plasma exposure (AUC_inf_) of the VD-deficient group increased slightly, there was no statistically significant difference in the pharmacokinetic parameters (C_max_ and AUC_inf_) between the groups (*p* > 0.05, Figures 4B,C). In addition, pravastatin concentration in liver tissue for the control group is 270.948 ± 206.56 ng/ml (*n* = 3), VD-deficient group is 146.94 ± 74.48 ng/ml (*n* = 3).

### Effect of Vitamin D Deficiency on the Oatps Expression in Rat Tissues

**FIGURE 5:**
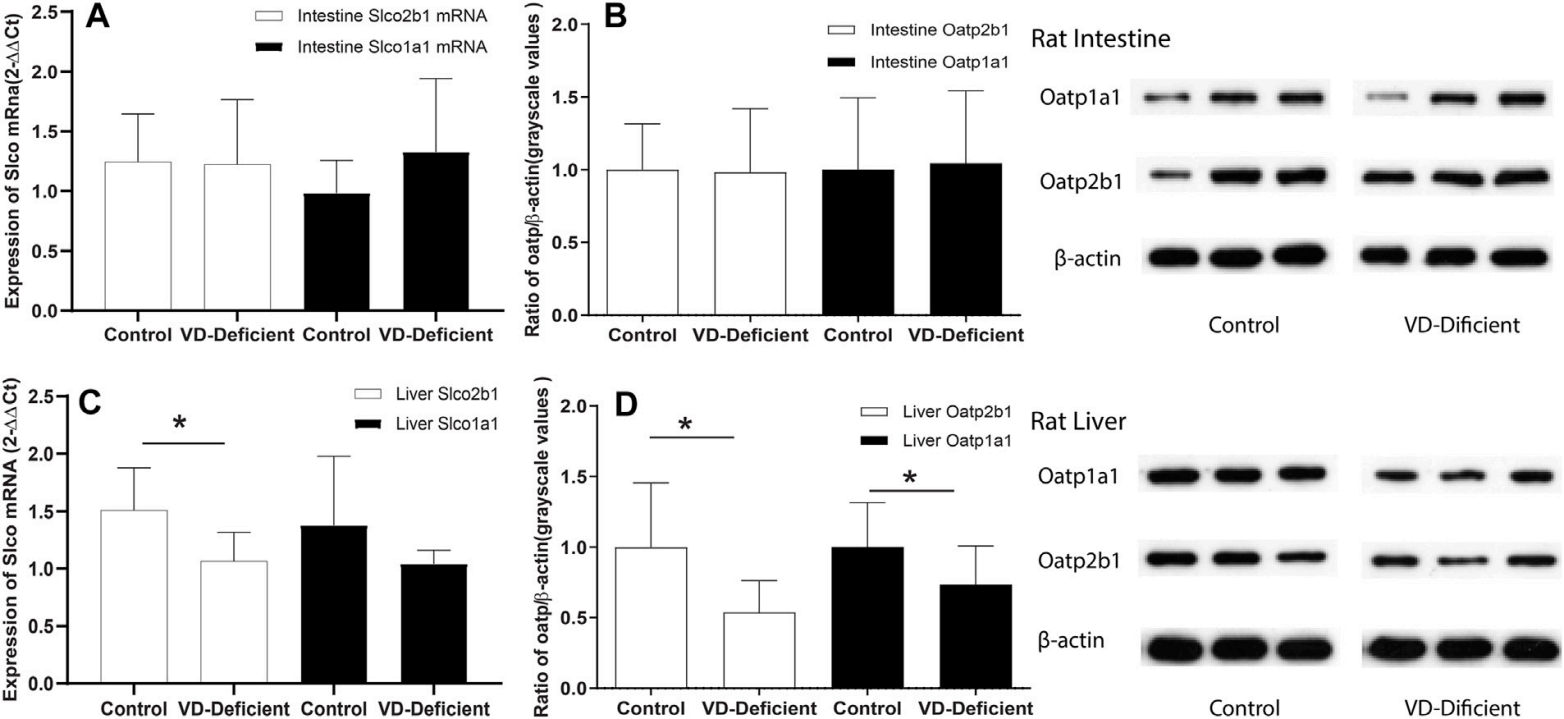
Effects of VD deficiency on the Oatps mRNA and protein expressions in rat tissues. **(A)** Slco2b1 and Slco1a1 mRNA expression (normalized by β-actin) in the rat intestine. **(B)** Oatp2b1 and Oatp1a1 protein expression in the rat intestine. **(C)** Slco2b1 and Slco1a1 mRNA expression (normalized by β-actin) in rat liver. **(D)** Oatp2b1 and Oatp1a1 protein expression in rat liver. VD deficiency can down-regulate Oatps expression in the liver but has no significant effect in the small intestine. 2^−△△Ct^ and grayscale values were used to compare genes and protein expressions between the groups, respectively, and β-actin was used as the reference gene. Control group: rats fed on a VD-supplement diet (*n* = 6), 25(OH)VD concentration is 20–100 ng/ml, VD-deficient: rats fed on a VD-free diet (*n* = 9), 25(OH)VD concentration is <20 ng/ml *p < 0.05: statistically significant difference, using two-tailed unpaired t-test.

qPCR and WB assays were performed to determine the Oatp2b1 and Oatp1a1 expression in rat liver and small intestine tissue, respectively, in the pharmacokinetic study. Results showed that Slco2b1, Slco1a1 mRNA, and Oatp2b1 and Oatp1a1 protein in rat intestine tissue were not affected by VD deficiency (Figures 5A,B). Expression of Slco2b1 and Slco1a1 mRNA in the liver of VD-deficient rats (*n* = 9) was lower than the Control (*n* = 6) (*p* = 0.035, 0.07), and the protein expression of Oatp2b1 and Oatp1a1 was reduced in VD-deficient rats (*p* = 0.005, *p* = 0.047, Figures 5C,D). VD deficiency did not affect the Oatps expression of rat small intestine but decreased their expression in the liver.

### Effects of Vitamin D Deficiency on OATPs in the Normal Liver Huh7 Cell Line

**FIGURE 6:**
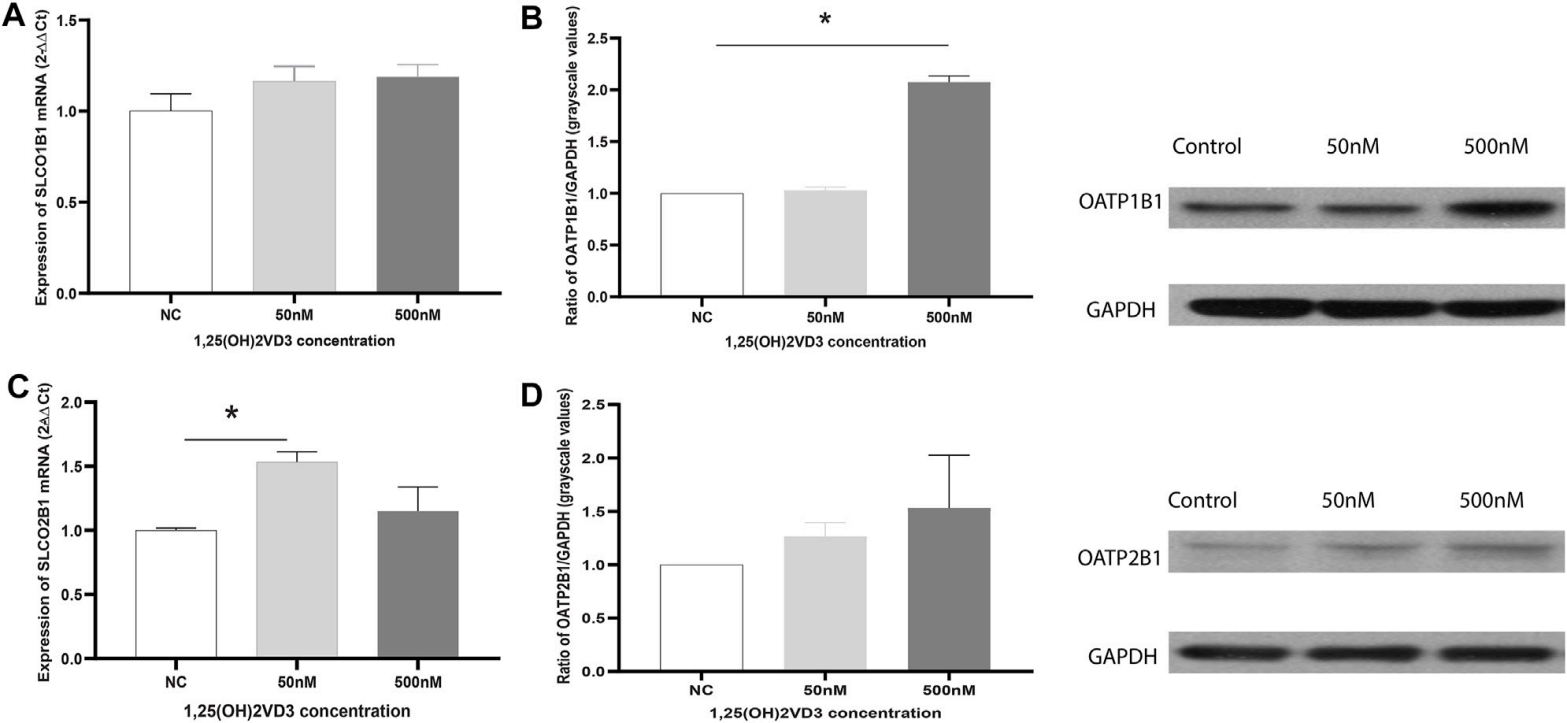
Effects of 1,25(OH)2VD3 on OATP1B1 and OATP2B1 expression in Huh7 cells. **(A)** SLCO1B1 mRNA expression. **(B)** OATP1B1 protein expression. **(C)** SLCO2B1 mRNA expression. **(D)** OATP12B1 protein expression. 2^−△△Ct^ and grayscale ratio values of OATPs and GAPDH were used to compare the genes and protein expression between the groups, respectively, and GAPDH was used as the reference gene. NC: Blank control (ethanol), 50 and 500 nM: different groups using 50 and 500 nM 1.25(OH)_2_VD_3_. *p < 0.05: statistically significant difference, using ANOVA followed by Tukey’s post-hoc test.

The effects of 1.25(OH)_2_VD_3_ on OATP2B1 and OATP1B1 in Huh7 cells were explored. OATP1B1 and OATP2B1 expression increased with 1.25(OH)_2_VD_3_ (Figure 6), indicating that the expression of OATP1B1 and OATP2B1 in the liver were lower when it is VD deficient, which is similar to that found in the rat liver.

## Discussion

In a previous study, we reported that VD may interact with some drugs (Peng et al., 2020). However, the impact of VD deficiency on drugs has not been clarified. In this study, we found that VD deficiency decreased the response of pravastatin (changes in blood lipids), slightly increased plasma exposure and decreased the liver exposure (*p* > 0.05). VD deficiency down-regulated Oatp1a1 and Oatp2b1 in the liver but did not significantly alter intestinal Oatp2b1 and Oatp1a1 expression in rats. In addition, 1.25(OH)_2_VD_3_ upregulated OATP1B1 and OATP2B1 expression in normal liver cell lines. To the best of our knowledge, this study is the first report of the effects of VD deficiency on pravastatin, OATP2B1, and OATP1B1.

VD deficiency decreased the response of pravastatin, which is rarely metabolized by CYP enzymes (Bhattacharyya et al., 2012). On the one hand, although the blood lipids of all rats were reduced due to decreased food consumption associated with gavage-induced stress (de Meijer et al., 2010), the TG and TC decreased less in VD-deficient rats than in the control group, and HDL increased less. Other authors report that VD deficiency is significantly associated with an increase in blood lipids and cardiovascular disease, and vitamin D_3_ supplementation could reverse these effects (Jastrzebski et al., 2016; Wang et al., 2016; Marisa et al., 2018; Kim and Jeong, 2019). However, in the present study, there is no significant difference between the two groups (Control vs. VD-deficient, *p* > 0.05), which may be due to the limited exposure time (25–30 days). On the other hand, in the present study, pravastatin more effectively lowered lipid levels in VD-sufficient rats than in VD-deficient rats. For the control groups (Control, Control + P), the pravastatin-induced change of TG, TC and HDL/TC (Control + P subtracting Control) were −17.40%, −9.62%, and 20.09%, respectively, which indicated that pravastatin improves the blood lipid situation in VD-sufficient rats. Quite the opposite, the pravastatin-induced change of TG, TC and HDL/TC in the VD-deficient groups (VD-deficient + P subtracting VD-deficient) were 10.10%, 7.06%, and −1.90%, respectively, which indicated that pravastatin did not produce lipid-lowering effects in the VD-deficient rats. This suggests that we should pay attention to patients’ plasma VD levels before using pravastatin in the clinic; when their VD levels are low, the response of pravastatin will be reduced or may even disappear. Additionally, changes in blood lipid of pravastatin-treated/-untreated control groups were not statistically significant, which may be due to the small sample size or the dose of pravastatin (10 mg/kg/day). Optimizing the dose of pravastatin or increasing the number of rats can make the difference between pravastatin-untreated and treated control rats statistically significant.

For the effects of VD deficiency on pharmacokinetics of pravastatin, the liver exposure of pravastatin decreased, plasma exposure increased slightly, and enterohepatic circulation (the second peak in Figure 4C) were attenuated in VD-deficient rats. Hepatic uptake activity (OATPs), not hepatic efflux (MRPs), plays an important role in the effects of VD deficiency. First, the extended liver clearance model theory (Patilea-Vrana and Unadkat, 2016) states that, for drugs eliminated by the kidney, when hepatic uptake clearance (OATPs mediated) decreases, the plasma concentration increases, and the hepatic drug concentration will reduce (Kusuhara and Sugiyama, 2009). Our results are consistent with this theory: VD deficiency decreased OATPs expression (Figures 5, 6); there was a trend towards increased plasma exposure (AUC, Figure 4) and decreased liver tissue; consequently, pravastatin response was reduced. Second, downregulation/unchanged of Mrp2 expression in the absence of VD should theoretically enhance pravastatin exposure in the liver (Fan et al., 2009; Ogura et al., 2009; Chow et al., 2010), which is inconsistent with the decline in liver pravastatin concentration under VD deficiency conditions. This indicates that MRP2 is not involved the effect of VD deficiency on pravastatin. Last but not least, pravastatin is transported by renal OAT3/Oat3 in humans and rats (Maki et al., 2002; Khamdang et al., 2004; Watanabe T. et al., 2011; Mathialagan et al., 2017). Activity and expression of OAT3 in rat and mouse kidneys are higher under VD deficiency (Kim et al., 2014; Quach et al., 2018), indicating that VD deficiency may increase the renal secretion of pravastatin and decrease pravastatin exposure. Reducing hepatic Oatps expression in VD-deficient rats causes an increase of pravastatin concentration in the plasma, which can be offset by changes of Oat3 in VD-deficient rats, further decreasing the response of pravastatin.

VD deficiency can significantly decrease OATPs/Oatps expression in the liver, but not in rat intestine. This may be caused by differences in tissues or the different distribution of 1.25(OH)_2_VD between the two tissues. The effect of 1.25(OH)_2_VD_3_ on OATP2B1 and OATP1B1 was confirmed in Huh7 cells and is consistent with the effect in rats. Low concentrations of 1.25(OH)_2_VD_3_ (10,100 nM) did not significantly induce OATP1B1 or OATP2B1 expression (data not shown), 50 nM (20.83 ng/ml) and 500 nM (208.30 ng/ml) 1.25(OH)_2_VD_3_ was used for *in vitro* experiments and it was higher than the plasma concentration of 25(OH)VD in control rats (20–100 ng/ml). Furthermore, the vitamin D receptor (VDR)-miRNA pathway may mediate the effect of VD deficiency on OATP2B1 (Oatp2b1) and OATP1B1 expression in the liver. In previous studies, VDR expression or activity was found to be inversely correlated with miRNAs (e.g., miRNA-346, miRNA-17/92, miRNA-155, miRNA-181, miRNA-302, and miRNA-520c) (Wang et al., 2009; Wang et al., 2011; Chen et al., 2013; Min et al., 2013; Wang et al., 2013; Li et al., 2014; Borkowski et al., 2015; Sheane et al., 2015; Yi et al., 2016). We and other researchers reported that miRNA-511 down-regulates the expression of OATP1B1 and OATP2B1 (Peng et al., 2015; Liu et al., 2020). Therefore, it is likely that VD may activate the VDR/miRNA pathway and hence upregulate OATP1B1 and OATP2B1 expression.

Our research has several limitations. Firstly, the concentration of pravastatin in the rat liver was measure at single time point (4 h post-dose), investigations in future should measure the concentration of drugs in the liver at multiple time points. Secondly, we didn’t explore whether pravastatin and VD deficiency jointly affect the expression of OATPs, but there is no study shown that pravastatin can affect the expression or activity of OATPs; 25(OH)VD2 is much lower than 25(OH)VD3, the effect of VD2 hasn’t been studied. We can only assume that they are negligible. Thirdly, using the diet method to control VD concentration in rats can result in significant differences in the concentration of VD in rats, because each rat may take a different quantity of the diet. Fourthly, the number of rats in this study was small, the increase in pharmacokinetics were not statistically significant. Fifthly, as fraction transported by OATP1B3 much lower than that by OATP1B1 (25 vs. 74%) and they are highly homologous (Alam et al., 2018; Bowman et al., 2021), the effect of VD on OATP1B3 hasn’t been studied. Finally, Oatp2b1 and Oatp1a4 may also be expressed in muscles and can affect pravastatin-induced myopathy (Sakamoto et al., 2008). However, in our study, no investigation was conducted on muscle transporters.

## Conclusion

VD deficiency decreased the response and liver exposure of pravastatin and increased the plasma exposure of pravastatin, but not significantly, which were mediated by reducing hepatic Oatp2b1 and Oatp1a1 expression. This study revealed the effect of VD deficiency on the pravastatin pharmacology and OATPs expression and can therefore provide reference data for the clinical management of patients with VD deficiency. The results presented here also imply that VD deficiency should also be considered when designing OATPs substrate drugs.

## Data Availability

The original contributions presented in the study are included in the article/Supplementary Material, further inquiries can be directed to the corresponding authors.

## Abbreviations

1.25(OH)2D, 1.25 dihydroxy vitamin D; 25(OH) D, 25-hydroxyvitamin D; AUCinf, area under the curve from the time of dosing extrapolated to infinity; Cmax, Maximum Plasma Concentration; cDNA, Complementary DNA; CYP, Cytochrome P450; ESI, Electrospray ionization source; GAPDH, Glyceraldehyde 3-phosphate dehydrogenase; HDL-C, high-density lipoprotein cholesterol; HMG-CoA, β-Hydroxy β-methylglutaryl-CoA; LC, liquid chromatography; LDL-C, Low-density lipoprotein cholesterol; miRNA, microRNA; mRNA, Messenger RNA; MRP, Multidrug resistance-associated protein; MS, tandem mass spectrometry; NCA, Noncompartmental PK Analysis; OAT, organic anion transporter; OATPs, Organic anion-transporting polypeptides; qPCR, quantitative polymerase chain reaction; SD rats, Sprague Dawley Rats; SLCO, solute carrier organic anion transporter; TC, total cholesterol; TG, triglyceride; WB, western blot; VD, Vitamin D; VDR, Vitamin D receptor.
