# Supplementary material for: Vitamin D Deficiency Impacts Exposure and Response of Pravastatin in Male Rats by Altering Hepatic OATPs
Source: Front Pharmacol. 2022 Feb 17;13:841954. doi: 10.3389/fphar.2022.841954 (PMC8892078; doi:10.3389/fphar.2022.841954)
Supplement: Supplementary file 1 [file Table1.docx]

**Vitamin D deficiency impacts exposure and response of pravastatin in male rats by altering hepatic OATPs**

Jinfu Peng ^1,2,*^, Guoping Yang ^2^, and Zhijun Huang ^2,3,*^

^1^ Department of Pharmacy, the Third Xiangya Hospital, Central South University, Changsha, Hunan, China

^2^ Center for Clinical Pharmacology, the Third Xiangya Hospital, Central South University, Changsha, Hunan, China

^3^ Department of Nephrology, The Third Xiangya Hospital, Central South University, Changsha, China

* Correspondence: Jinfu Peng (+8618874102334), and Zhijun Huang (+8613908472564), Center for Clinical Pharmacology, the Third Xiangya Hospital, Central South University, 138 TongZiPo Road, Changsha, Hunan, 410013, China

E-mail: pengjinfu@csu.edu.cn, [huangzj@csu.edu.cn](mailto:huangzj@csu.edu.cn)

**Supplementary Table.** **Ingredients and contents of feeds**

| Feed | Ingredients | Weight (g/kg feed) |
| --- | --- | --- |
| VD- free high-fat feed | Casein | 0.175 |
|  | L-cystine | 0.0025 |
|  | Corn starch | 0.350 |
|  | Maltodextrin | 0.115 |
|  | Sucrose | 0.088 |
|  | Cellulose | 0.044 |
|  | Soybean oil | 0.062 |
|  | Choline bitartrate | 0.002 |
|  | AIN93G mineral supplement | 0.031 |
|  | vitamin mixture (VD deficiency) | 0.009 |
|  | antioxidant | 0.013 |
|  | cholesterol | 0.020 |
|  | lard | 0.100 |
| VD- supplement high-fat feed | Casein | 0.175 |
|  | L-cystine | 0.0025 |
|  | Corn starch | 0.350 |
|  | Maltodextrin | 0.115 |
|  | Sucrose | 0.088 |
|  | Cellulose | 0.044 |
|  | Soybean oil | 0.062 |
|  | Choline bitartrate | 0.002 |
|  | AIN93G mineral supplement | 0.031 |
|  | vitamin mixture (1.1mg VD_3_/kg feed) | 0.009 |
|  | antioxidant | 0.013 |
|  | cholesterol | 0.020 |
|  | lard | 0.100 |
| VD- free feed | Casein | 0.2 |
|  | L-cystine | 0.003 |
|  | Corn starch | 0.397 |
|  | Maltodextrin | 0.132 |
|  | Sucrose | 0.1 |
|  | Cellulose | 0.05 |
|  | Soybean oil | 0.07 |
|  | Choline bitartrate | 0.0025 |
|  | AIN93 mine Substance | 0.035 |
|  | vitamin mixture (VD deficiency) | 0.01 |
| VD- supplement feed | Casein | 0.2 |
|  | L-cystine | 0.003 |
|  | Corn starch | 0.397 |
|  | Maltodextrin | 0.132 |
|  | Sucrose | 0.1 |
|  | Cellulose | 0.05 |
|  | Soybean oil | 0.07 |
|  | Choline bitartrate | 0.0025 |
|  | AIN93 mine Substance | 0.035 |
|  | vitamin mixture (1.1mg VD_3_/kg feed) | 0.009 |
