# Supplementary figures and images for: Vitamin D Deficiency Impacts Exposure and Response of Pravastatin in Male Rats by Altering Hepatic OATPs
Source: Front Pharmacol. 2022 Feb 17;13:841954. doi: 10.3389/fphar.2022.841954 (PMC8892078; doi:10.3389/fphar.2022.841954)

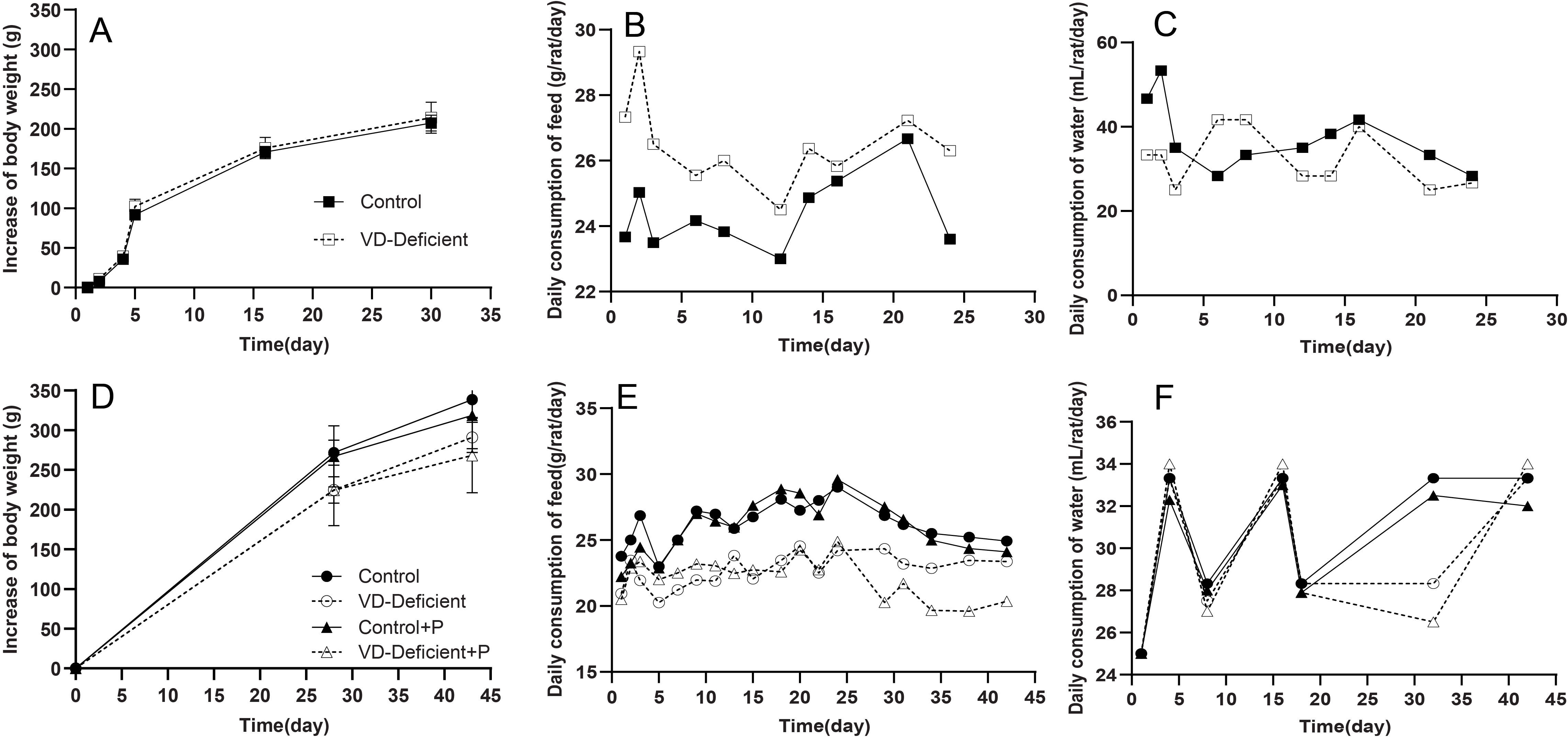

Supplement: Supplementary file 2 [file Image1.JPEG]
