# Supplementary material for: Vitamin D Deficiency Impacts Exposure and Response of Pravastatin in Male Rats by Altering Hepatic OATPs
Source: Front Pharmacol. 2022 Feb 17;13:841954. doi: 10.3389/fphar.2022.841954 (PMC8892078; doi:10.3389/fphar.2022.841954)
Supplement: Supplementary file 3 [file DataSheet1.docx]

**Vitamin D deficiency impacts exposure and response of pravastatin in male rats by altering hepatic OATPs**

Jinfu Peng ^1,2,*^, Guoping Yang ^2^, and Zhijun Huang ^2,3,*^

^1^ Department of Pharmacy, the Third Xiangya Hospital, Central South University, Changsha, Hunan, China

^2^ Center for Clinical Pharmacology, the Third Xiangya Hospital, Central South University, Changsha, Hunan, China

^3^ Department of Nephrology, The Third Xiangya Hospital, Central South University, Changsha, China

* Correspondence: Jinfu Peng (+8618874102334), and Zhijun Huang (+8613908472564), Center for Clinical Pharmacology, the Third Xiangya Hospital, Central South University, 138 TongZiPo Road, Changsha, Hunan, 410013, China

E-mail: pengjinfu@csu.edu.cn, huangzj@csu.edu.cn

**Supplementary file. LC-MS/MS methods for *25(OH)VD* and pravastatin**

***25(OH)VD_2_ and 25(OH)VD_3_***

Standard samples were obtained by mixing 100 µL of 4% BSA solution (PBS buffer) with 10 µL of the standard 25(OH)VD [25OHVD_2_ (Sigma, FN08051401, 99.6%), 25OHVD_3_ (Sigma, FN09111405, 100%)] curve working solution (25, 50, 100, 200, 400, 800, 1000, 2000ng/mL) or (50, 400, 1000ng/ml) quality control sample solution. Adding 10 µl of the internal standard solution [d6-25OHVD_2_ (1 µg/mL, Toronto Research Chemicals Inc, H995822, 98%), d6-25OHVD_3_ (1 µg/mL, Sigma, FN11141404, 99.54%)], 100 µL of the standard samples or rat plasma samples were taken for examinations. They were mixed for 1 min, then 25μl of 0.2M ZnSO4 and 300 µl of methanol were added, vortexed for 3 min, and stood for 10 min. 750 µl of n-hexane were then added and centrifuged (13000 rpm, 5 min) after shaking for 5 min. 700 µL of supernatant was transferred to a new EP tube and dried with nitrogen at room temperature. The remaining content was dissolved in 70 µl of 60% methanol-water and 10 µl added to a Waters Acquity UPLC system connected to a Xevo Triple Quadrupole Mass Spectrometer (UPLC/TQD-MS; Waters, Manchester, UK) for analysis.

The analytical column was AcQuITY UPLC® BEH HILIC, 50mm×2.1mm, 1.5μm (part NO. 186003460, serial NO.03003427918261 with a guard column (C18, 4 mm×2.0 mm, Phenomenex, Torrance, CA, USA). Mobile phase was aqueous (5mM ammonium acetate solution, containing 0.1% formic acid, A): methanol (0.1% formic acid, B). Liquid gradient elution conditions were: 0.00 min, 27:73; 2.00 min, 27:73; 3.50 min, 2 :98; 3.51, 27:73; 6.00 min, 27:73. The flow rate and the column temperature were 0.4 mL/min and 45°C, respectively.

Mass spectrometry ion source parameters included: electrospray ionization ESI source as the ionization source, the dissolvent gas temperature of 400℃, ion source temperature of 120℃, a capillary voltage of 2500V, and a chosen positive ion multiple reaction monitoring (MRM) mode. 25OHD_3_ monitoring ion conditions were m/z 401.3→m/z 365.3, cone voltage of 24.0 V, and collision energy of 10.0 V. 25OHD_2_ monitoring ion conditions were m/z 413.3→m/337.3, cone voltage of 24.0 V, and collision energy of 10.0 V. d6-25OHD_3_ monitoring ion conditions were m/z 407.3→m/z 371.3, cone voltage of 24.0 V, and collision energy of 28.0 V. d6-25OHD_2_ monitoring ion conditions were m/z 419.0→m/337.3, cone voltage of 24.0 V, and collision energy of 10.0 V.

***Pravastatin***

Liver tissues were homogenized at the ratio of 200 mg of liver and 200 μl of water. Standard samples were obtained by mixing 100 μl of blank SD rat blank plasma or 200 μl blank liver homogenous with 10 µl or 20 µl of pravastatin standard curve working solution (5, 12.5, 25, 50, 125, 250, 500ng/mL) or (12.5, 50, 250ng/mL) quality control sample solution. 10 µl or 20 µl of the internal standard solution [ibuprofen, 100 µg/mL, National Institutes for Food and Drug Control of China, 100179-201406, 100%],10 µL of 10% (V/V) formic acid was added to samples and shook for 3 min, then 1 mL of ethyl acetate added for extraction (3 min), then centrifuged (13000 rpm, 10 min). Extraction with 300 µL of ethyl acetate was repeated. The organic phase was combined and dried with nitrogen. A 50 µL mixture of methanol (0.1% formic acid) and 10mM ammonium acetate aqueous solution (0.05% acetic acid) (70:30) were used to dissolve, and 10 µL injected into a liquid chromatography system (Agilent 1260, USA) connected to a triple quadrupole mass spectrometer (Agilent 6460, USA) for analysis.

Eclipse XDB-C18 (2.1 mm×150 mm, 5 µm, Agilent, USA, PN 993700-902, SN usnm 005792) was used as the analytical column and methanol (0.1% formic acid):10 mM ammonium acetate aqueous solution (0.05% acetic acid) (70:30) as the mobile phase. The flow rate and the column temperature were 0.53 mL/min and 35°C, respectively.

Electrospray ionization source (ESI) and negative ion MRM mode were used. Pravastatin sodium monitoring ion conditions were m/z 423.1→m/z 320.9, cone voltage of 150 V, and collision energy of 12 V. Internal standard ibuprofen monitoring ion conditions were m/z 204.8→m/z 161.0, cone voltage of 75 V, and collision energy of 2 V. The ion source parameters were Gas Temp:350 ℃, Gas Flow:12 L/min Nebulizer,18.0 psi, Capillary voltage:4000 V.
